# Characterisation and Expression of Osteogenic and Periodontal Markers of Bone Marrow Mesenchymal Stem Cells (BM-MSCs) from Diabetic Knee Joints

**DOI:** 10.3390/ijms25052851

**Published:** 2024-03-01

**Authors:** Nancy Hussein, Josephine Meade, Hemant Pandit, Elena Jones, Reem El-Gendy

**Affiliations:** 1 Division of Oral Biology, School of Dentistry, University of Leeds, Leeds LS9 7TF, UK; dnnmsh@leeds.ac.uk (N.H.); j.l.meade@leeds.ac.uk (J.M.); 2 Department of Oral Medicine and Periodontology, Faculty of Dentistry, Mansoura University, Mansoura 35516, Egypt; 3 Leeds Institute of Rheumatic and Musculoskeletal Medicine, School of Medicine, University of Leeds, Leeds LS9 7TF, UK; h.pandit@leeds.ac.uk (H.P.); e.jones@leeds.ac.uk (E.J.); 4 Department of Oral Pathology, Faculty of Dentistry, Suez Canal University, Ismailia 41522, Egypt

**Keywords:** diabetes, bone marrow mesenchymal stem cells, bone regeneration

## Abstract

Type 2 diabetes mellitus (T2DM) represents a significant health problem globally and is linked to a number of complications such as cardiovascular disease, bone fragility and periodontitis. Autologous bone marrow mesenchymal stem cells (BM-MSCs) are a promising therapeutic approach for bone and periodontal regeneration; however, the effect of T2DM on the expression of osteogenic and periodontal markers in BM-MSCs is not fully established. Furthermore, the effect of the presence of comorbidities such as diabetes and osteoarthritis on BM-MSCs is also yet to be investigated. In the present study, BM-MSCs were isolated from osteoarthritic knee joints of diabetic and nondiabetic donors. Both cell groups were compared for their clonogenicity, proliferation rates, MSC enumeration and expression of surface markers. Formation of calcified deposits and expression of osteogenic and periodontal markers were assessed after 1, 2 and 3 weeks of basal and osteogenic culture. Diabetic and nondiabetic BM-MSCs showed similar clonogenic and growth potentials along with comparable numbers of MSCs. However, diabetic BM-MSCs displayed lower expression of periostin (POSTN) and cementum protein 1 (CEMP-1) at Wk3 osteogenic and Wk1 basal cultures, respectively. BM-MSCs from T2DM patients might be suitable candidates for stem cell-based therapeutics. However, further investigations into these cells’ behaviours in vitro and in vivo under inflammatory environments and hyperglycaemic conditions are still required.

## 1. Introduction

Type 2 diabetes mellitus (T2DM) is a metabolic disorder resulting from a lack of insulin cellular response (insulin resistance) leading to subsequent hyperglycaemia [1]. Increased bone fragility and higher fracture risk are common complications in patients with long-standing T2DM [2]. T2DM is an established risk factor for a number of comorbidities including cardiovascular disease [3,4], osteoarthritis [5,6] and osteoporosis [7]. Periodontitis is one of the most widespread inflammatory diseases among adults, with its advanced forms affecting 10–15% of the population globallyIn its later stages, it can lead to the subsequent destruction of the toth attachment apparatus (periodontium) that supports and anchors teeth within the jaw bone [8]. T2DM is also a risk factor for periodontitis where the morbidity and severity of periodontitis in diabetics positively correlate with poorly controlled or long-standing diabetes [8].

Furthermore, enhancing bone regeneration is required in complicated clinical scenarios including larger defects beyond the physiologic potential of bone self-healing such as traumatic injuries and tumour resection or cases in which these regenerative potentials are undermined, such as patients with systemic disease [9].

Furthermore, nonsurgical periodontal therapy offers limited if any restoration of the form or function of periodontal tissues [10].

Stem cell-based dental and skeletal tissue regeneration is a promising therapeutic approach [11], and bone marrow mesenchymal stem cells (BM-MSCs) are one of the most investigated MSCs for bone regeneration due to their superior osteogenic capabilities [12,13,14,15,16]. BM-MSCs have been through several clinical trials for treating knee osteoarthritis where they improved clinical and radiographic outcomes [17,18,19,20]. Bone regeneration using BM-MSCs was also the subject of multiple clinical trials [21], including a phase I/II study where autologous BM-MSCs were transplanted to infrabony periodontal defects and led to improved probing depth, tooth mobility and linear bone growth without safety issues [22]. Autologous BM-MSCs in particular offer multiple advantages over other approaches for bone regeneration; there is no need for donor recruitment as they are isolated from the same patient with minimal or no risk of immunologic reaction associated with allogenic cell transplantation and thus no need for subsequent immunosuppressive therapy [23]. However, evidence on the impact of T2DM on BM-MSCs is still inconclusive [23]. While some studies reported similar mineral deposition by diabetic (D) and nondiabetic (ND) BM-MSCs [24,25], others described inferior osteogenic potentials [26]. The aim of the current study was to determine whether T2DM had an influence on stem cell characters and the expression of osteogenic and periodontal markers, including periostin (POSTN) and cementum protein 1 (CEMP-1), of BM-MSCs from knee joints of T2DM patients. POSTN is a marker of PDL fibroblast differentiation [27] and was shown to induce periodontal tissue regeneration [28]. CEMP-1 is a marker of cementoblast differentiation [29], with its derived peptides enhancing periodontal regeneration in animal models [30].

## 2. Results

### 2.1. Colony Formation and Proliferation

**Figure 1:**
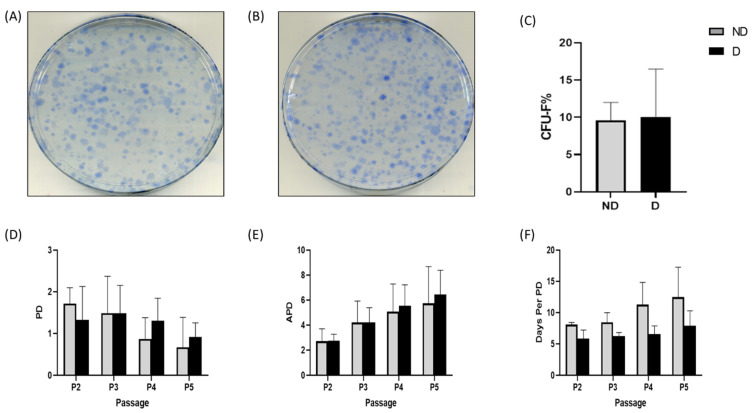
CFUF-Fs and PDT assays of nondiabetic (ND) and diabetic (D) BM-MSCs. (**A**) Representative CFU-Fs of early-passage BM-MSCs isolated from an ND and (**B**) a D donor. (**C**) CFU-Fs% in ND and D donors. (**D**) Population doubling, (**E**) accumulative population doubling and (**F**) population doubling time in ND and D BM-MSCs. Data are presented as mean ± SD (*n* = 3) and analysed using unpaired t test.

In the colony-forming unit fibroblast (CFU-F) assay, colonies were counted after 14 days of culture of D and ND BM-MSCs (each *n* = 3). The mean number of CFU-Fs in ND cells was 9.58 ± 2.42%, while D BM-MSCs on average had 10.07 ± 6.41% CFU-Fs, and this difference was not statistically significant (*p* = 0.91) (Figure 1). For the population doubling time (PDT) assay, population doubling (PD), accumulative population doubling (APD) and PDT of ND vs. D BM-MSCs at passages (Ps) 2–5 showed no statistically significant differences (Figure 1). Details of PD, APD and PDT of non-diabetic (ND) and diabetic (D) BM-MSCs are in Supplementary Data Table S1.

### 2.2. Expression of MSCs’ Cell Surface Markers

**Figure 2:**
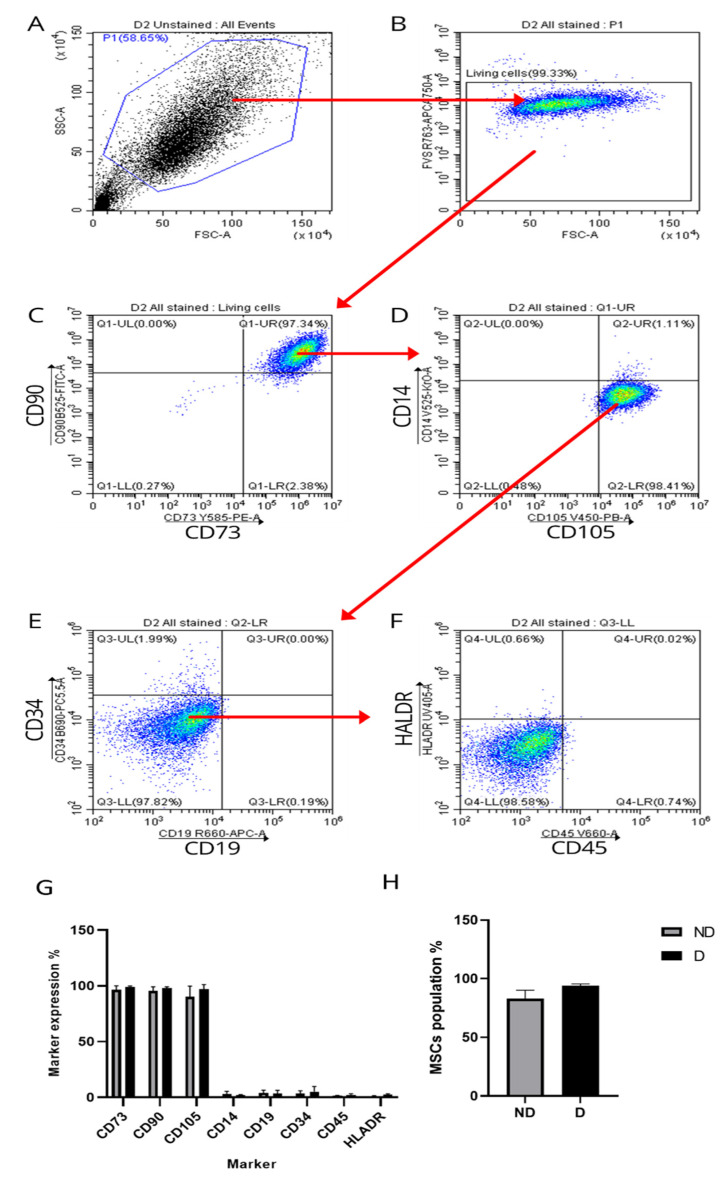
Enumeration of MSC population in diabetic (D) BM-MSCs. (**A**) Gating to exclude dead cells and debris based on their forward and side scatter. (**B**) Gating to include living cells based on their negative uptake of fixable viability stain. (**C**) Gating to include CD73^+^CD90^+^ cells in quadrant Q1-UR. (**D**) Gating to include CD73^+^CD90^+^CD105^+^CD14^−^ cells in quadrant Q2-LR. (**E**) Gating to include CD73^+^CD90^+^CD105^+^CD14^−^CD19^−^CD34^−^ in quadrant Q3-LL. (**F**) Gating to include CD73^+^CD90^+^CD105^+^CD14^−^CD19^−^CD34^−^CD45^−^HLA-DR^−^ in quadrant Q4-LL. The same approach was employed with BM-MSCs from nondiabetic (ND) and D donors. (**G**) Expression of MSC markers in ND and D BM-MSCS. (**H**) Enumeration of MSC population in ND and D MSCs. Data are presented as mean ± SD (*n* = 3) and analysed using unpaired *t* test.

The majority of cells in cultures of ND and D BM-MSCs expressed CD73, CD90 and CD105 and lacked expression of CD14, CD19, CD34, CD45 and HLA-DR (Figure 2). As defined by the ISCT [31], the MSC population was quantified following sequential gating using the immunophenotype CD73^+^/CD90^+^/CD105^+^/CD14^−^/CD19^−^/CD34^−^/CD45^−^/HLA-DR^−^ (Figure 2), as previously described [32]. The average MSC population in ND cells was 82.88 ± 7.28%, while D cells contained 94.02 ± 1.5% MSCs, and this difference was not statistically significant (*p* = 0.11) (Figure 2).

### 2.3. Osteogenic Differentiation of ND and D BM-MSCs

#### 2.3.1. Alkaline Phosphatase (ALP) Staining

**Figure 3:**
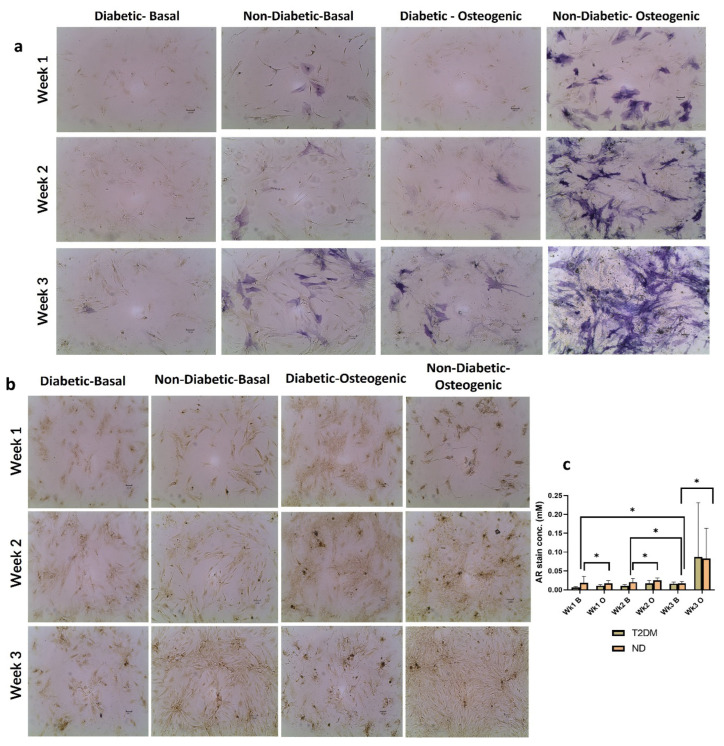
Comparing osteogenic differentiation of diabetic (D) and nondiabetic (ND) BM-MSCs: (**a**) Alkaline phosphatase stain of D and ND BM-MSCs under basal and osteogenic conditions after 1, 2 and 3 weeks of culture. (**b**) Alizarin Red (AR) stain of ND and D BM-MSCs under basal and osteogenic conditions after 1, 2 and 3 weeks of culture. (**c**) Quantification of AR stain of ND and D BM-MSCs. Data are presented as mean ± SD (*n* = 3), analysed using unpaired *t* test and showed no significant difference (* *p* < 0.05)).

ALP under basal conditions, both in ND and D samples, showed weak staining, except at week 3, where the ND samples showed a slight increase in the stain, whereas, under osteogenic conditions, there is clear evidence of osteogenic progression at all time points for ND samples and only at week 3 for D samples. The overall staining intensity was higher in ND BM-MSCs compared to D BM-MSCs (Figure 3a).

#### 2.3.2. Alizarin Red Staining (ARS)

ARS indicated a similar pattern to that of ALP with weaker overall staining in both cell populations (Figure 3b). This was reflected in the quantitative data, which showed no significant difference in ARS quantification between the two cell populations, under the two different culture conditions at all time points (Figure 3c). However, AR staining quantification showed a statistically significant higher mineralization at Wk 3 under basal cultures of D BM-MSCs in comparison to Wk1 (*p* < 0.05) and Wk2 (*p* < 0.05). D cells also showed a statistically significant higher staining under osteogenic conditions in comparison to basal culture controls at Wk1 (*p* < 0.05) and Wk2 (*p* < 0.05), and a similar difference was observed between ND cells cultured under basal and osteogenic conditions at Wk3 (*p* < 0.05) (Figure 3c).

#### 2.3.3. Gene Expression

**Figure 4:**
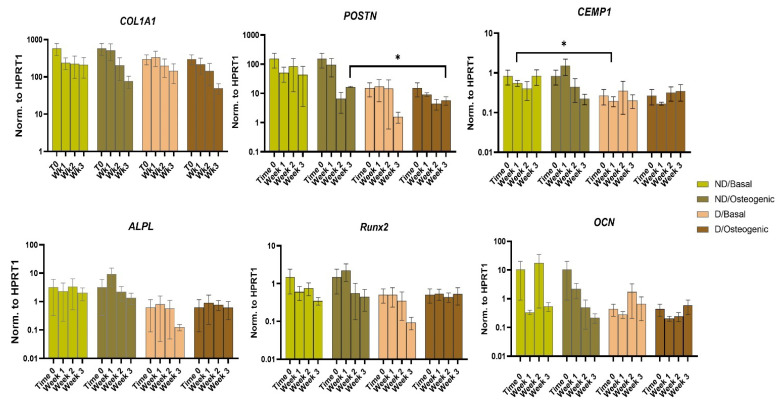
Comparing the relative changes in gene expression of periodontal markers (top panel: COL1A1, POSTN and CEMP-1) and osteogenic markers (lower panel: ALPL, Runx2, OCN) in ND and D BM-MSCs cultured under basal or osteogenic media for 1, 2 and 3 weeks as well as baseline untreated cells (0 time point). Data are presented as mean value ± SEM (*n* = 3) normalised to housekeeping (HPRT1) and were statistically analysed using unpaired *t* test for unmatched groups, paired *t* test for matched groups and repeated measures ANOVA for comparing time points, * *p* < 0.05.

Expression of periodontal markers: COL1 showed a trend of downregulation in Wk3 osteogenic vs. basal cultures of both ND and D BM-MSCs without significant differences between both cell populations at any time point or culture condition. POSTN expression was significantly lower in D vs. ND cells in Wk3 osteogenic cultures (*p* < 0.05). CEMP-1 expression was significantly lower in D vs. ND BM-MSCs in Wk1 basal cultures (*p* < 0.05) (Figure 4). For all genes, comparing time points using repeated measures ANOVA (RM-ANOVA) showed no time-dependent changes.

Expression of osteogenic markers genes ALPL, RUNX2 and OCN was comparable in both D and ND cells under both basal and osteogenic conditions with no statistically significant differences observed. However, there was a trend of lower ALPL expression in D vs. ND BM-MSCs at Wk3 basal cultures (Figure 4). ALPL also showed a trend of upregulation in osteogenic vs. basal cultures of ND BM-MSCs at Wk1 and D BM-MSCs at both Wk1 and Wk3. There was also a trend of RUNX2 upregulation in osteogenic vs. basal cultures in both D and ND BM-MSCs albeit at different time points. For ND cells, this upregulation was observed at Wk1, whereas for D cells, it was detected at Wk3 (Figure 4). 

**Figure 5:**
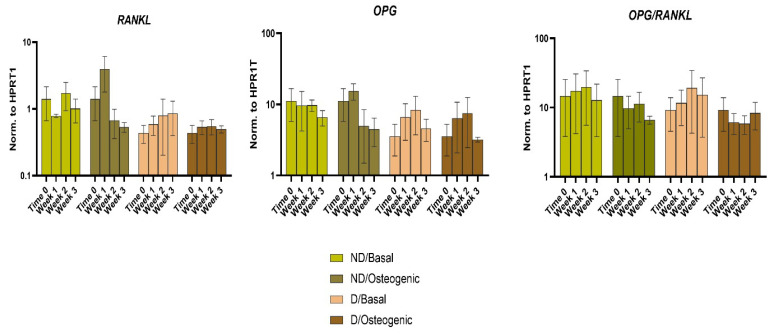
Comparing the relative changes in gene expression of bone homeostasis markers: RANKL, OPG, OPG/RANKL ratio) in ND and D BM-MSCs cultured under basal or osteogenic media for 1, 2 and 3 weeks as well as baseline untreated cells (0 time point). Data are presented as mean value ± SEM (*n* = 3) normalised to housekeeping (HPRT1) and were statistically analysed using unpaired *t* test for unmatched groups, paired *t* test for matched groups and repeated measures ANOVA for comparing time points. There was no significant difference between D and ND BMSCS, culture conditions or time points (NS > 0.05).

OPG and RANKL expression levels showed no statistically significant differences between ND and D cells at any time point or culture condition. The OPG/RANKL ratio was above 1 for both cell populations at different time points, indicating a higher osteoblastic activity but without significant differences between D and ND cells (Figure 5). 

## 3. Discussion

This study represents—to the best of our knowledge—a first attempt to characterize BM-MSCs isolated from the knee joints of T2DM patients, including their clonogenic, proliferative and osteogenic differentiation potentials. Moreover, this study would also be the first to investigate the expression of periodontal marker genes POSTN and CEMP-1 in D BM-MSCs.

D and ND BM-MSCs showed similar percentages of CFU-Fs (Figure 1), which agrees with the previous literature [33,34]. However, Cassidy et al. [25] reported reduced CFU-Fs in D BM-MSCs, possibly because they isolated BM-MSCs from bone marrow aspirate of femoral necks during hip replacement surgery of relatively younger donors compared to our study. It is thus possible that an age-related decrease in CFU-Fs as reported in earlier studies [35,36] has masked any potential changes related to the D status of the donors in our study. Both cell populations showed comparable proliferation rates as shown by PDT assay (Figure 1), which is consistent with other reports [24,25,34,37]. In contrast, BM-MSCs cultured under high glucose (HG) [38] or other diabetes simulation conditions such as a combination of HG and palmitic acid [39] or advanced glycated endproducts (AGEs) [40] showed significantly lower proliferation capacities. Such studies usually use nonphysiological high concentrations of glucose [41] and may not fully replicate the T2DM microenvironment that entails hyperglycaemia as well as high levels of AGEs, reactive oxygen species (ROS), hyperlipidaemia, hyperinsulinemia and inflammatory cytokines [42].

ND and D cells included comparable numbers of MSCs, similar to findings reported by Gabr et al. [37], who isolated D BM-MSCs from bone marrow aspirate of hip joints, which requires no enzymatic digestion to release the cells. Thus, it is unlikely that the harvesting site or isolation technique would influence the expression patterns of MSC surface markers. The presented results are also in agreement with those reported by Andrzejewska et al. [24] and Cassidy et al. [25], where both D and ND BM-MSCs presented the MSC phenotype reported in our results with no significant differences.

Higher ALP staining intensity was observed in cultures of ND vs. D BM-MSCs (Figure 3), which agrees with the work of Sun et al. [26] and Liang et al. [43], who reported lower ALP staining and activity in cultures of alveolar bone BM-MSCs isolated from D vs. ND donors. Thus, D BM-MSCs potentially show lower levels of ALP activity despite variation in the anatomical source. Matrix mineralization assessed by quantification of Alizarin Red stain was similar in D and ND cells, which agrees with the findings of Andrzejewska et al. [24] and Cassidy et al. [25]. In contrast, other investigations concluded that cultures of alveolar bone BM-MSCs from D donors had lower AR staining compared to ND cells [26,43,44], suggesting that calcium deposition by alveolar bone BM-MSCs could be more prone to be affected by diabetes compared to knee joint BM-MSCs that were used in the present study.

Our data showed no difference in COL1 expression between D and ND BM-MSCs at any time point (Figure 4). Conversely, D BM-MSCs isolated from the alveolar bone [26] and osteoblast cultures supplemented with AGEs both displayed COL1 downregulation [45]. Our data also indicated that both D and ND cells displayed a trend of COL1 downregulation in Wk3 osteogenic cultures, similar to earlier reports of osteogenic cultures of adipose tissue mesenchymal stem cells at days 10 and 18 [46]. A possible explanation is that COL1 represents an early marker of osteoblast differentiation (usually peaking at day 4–7 of culture) and an essential constituent of the initial organic phase of calcified tissues ECM [47].

POSTN expression levels in D BM-MSCs were significantly lower than ND cells in Wk3 osteogenic cultures (*p* < 0.05) (Figure 4). PDL cells cultured under AGEs did show POSTN downregulation at both gene and protein levels [48]. On the other hand, Seubbuk et al. [49] reported POSTN upregulation in PDL cells cultured under HG conditions. POSTN is upregulated in healing bone following fracture [50] and could enhance migration, proliferation and differentiation of periodontal ligament stem cells (PDLSCs) [51] as well as viability and adhesion of osteoblast-like cells [52]. Thus, these lower POSTN expression levels reported in the present study are a point to consider if autologous diabetic BM-MSCs were to be used as a regenerative therapeutic modality for bone and periodontal defects. Interestingly, POSTN can reverse the negative impact of HG [53] and AGEs [48] on the osteogenic potentials of PDLSCs, suggesting a protective role in the D microenvironment of the periodontium. 

CEMP-1 showed statistically significant lower expression in D vs. ND cells in Wk1 basal cultures (*p* < 0.05) (Figure 4). To the best of our knowledge, this marker has not been investigated in D BM-MSCs or BM-MSCs cultured under D conditions before, so there are not yet any close comparisons. In fact, little is known about the cementoblastic differentiation of BM-MSCs [54] compared to PDLSCs or dental stem cells in general. Nevertheless, CEMP-1 was downregulated at the protein level in PDL cells cultured under HG conditions in a time-dependent manner [55]. Reduced cementum thickness was noted in extracted teeth of D vs. ND subjects [56]. With reports of CEMP-1 (or its derived peptides) inducing osteogenic differentiation of gingival fibroblasts [57], oral mucosal stem cells [58] and PDL cells [29], it seems CEMP-1 functions extend beyond being a marker of cementoblastic differentiation and could be a key molecule for inducing bone and periodontal regeneration both in healthy models and under D conditions.

Expression levels of osteogenic markers genes ALPL, RUNX2 and OCN were generally similar in D and ND BM-MSCs regardless of culture media or time point (Figure 4). This contrasts the work of Sun et al. [26], who reported lower expression levels of RUNX2 and OCN, and Liang et al. [43], who described lower expression of OCN in D alveolar bone BM-MSCs. Our data are also in contrast with the findings of Ying et al. [59], who cultured BM-MSCs under osteogenic and HG osteogenic conditions and found that HG cultures caused ALPL downregulation. Healthy BM-MSCs cultured under HG [59], HG and palmitic acid [39] and T2DM serum [60] showed lower expression of both RUNX2 and OCN compared to control cultures. Nonetheless, these diabetes simulation cultures have some limitations, as mentioned above.

The data presented here showed that ALPL gene expression had a trend of upregulation under osteogenic vs. basal media at Wk1 in both D and ND cells, which agrees with the work of Liu et al. [61] This mirrors the differences observed in ALP staining intensity and is consistent with ALP being an essential early marker of active osteogenesis that is usually upregulated during the initial phases of osteogenic differentiation [62]. The ALP enzyme induces hydrolysis of ATP and pyrophosphate and is thus necessary for phosphate production and hydroxyapatite crystallization [63]. Still, this ALPL upregulation attributed to the effect of osteogenic culture of D cells was only notable at Wk3, indicating a possible later ‘rescuing’ effect of osteogenic supplements on ALPL expression.

The ALP staining intensity on the other hand peaked at Wk3 osteogenic culture, as it takes time for the ALP enzyme to be produced and accumulated at a level where activity can be detected.

RUNX2 is a transcription factor and hence is expressed early during osteogenesis, as it guides MSCs to differentiate into osteoblasts and inhibits their adipogenic differentiation. Furthermore, RUNX2, along with Osterix and the canonical Wnt pathway, controls the transition of osteoblast progenitors into immature osteoblasts, expressing osteogenic markers such as COL1 [64]. This could explain the present findings, where RUNX2 showed a trend of upregulation under osteogenic cultures of ND BM-MSCs at Wk1 and in D cells at Wk3. This is supported by reports on the early upregulation of RUNX2 during osteogenic differentiation of healthy BM-MSCs [61], and the later upregulation in D BM-MSCs at Wk3 possibly indicates a relatively weaker and slower response of D cells to osteogenic induction.

While RANKL/RANK binding modulates osteoclast differentiation and activation, OPG prevents excessive bone resorption by binding to RANKL, thus inhibiting its binding to RANK. This is why the OPG/RANKL ratio can be used as a predictor of bone mass [65]. The presented data showed that OPG expression rates were higher compared to RANKL with OPG/RANKL ratios above 1, implying that bone deposition was favoured over bone resorption in both D and ND BM-MSCs (Figure 5), with no significant differences of OPG, RANKL expression or their ratio in D vs. ND cells. Zhang et al. [66] described both OPG downregulation and RANKL upregulation, while Wu et al. [67] and Feng et al. [68] reported unchanged OPG expression and RANKL upregulation in PDL cells cultured under HG. RANKL upregulation was also observed in PDL cells cultured under HG and TNF-α [69] and in osteoblast cultures supplied with AGEs [45].

Although inducing osteogenic differentiation for 3 weeks has been described previously with different cell types including BM-MSCs, [70,71], DPSCs [32] and PLSCs [72], extending the osteogenic induction to 4 weeks in future work could provide a better insight regarding the osteogenic potentials of diabetic MSCs.

Moreover, the relatively old age and osteoarthritic status of the donors included in this study could have had a negative impact on the osteogenic differentiation of both D and ND BM-MSCs. Nonetheless, the main aim of the present study was to investigate the possible impact of T2DM on the osteogenic potentials of BM-MSCs. 

We recognize that having a relatively small sample size (*n* = 3 for each group) is a limitation of this study. However, trends of differences were successfully observed between D and ND cells, for instance, the interesting trends observed in gene expression of BM-MSCs in this study. This could have possibly reached statistical significance with a larger sample size. However, this limitation does not prevent us from sharing these data as an example of comprehensive analysis to guide future studies in the field. 

## 4. Materials and Methods

### 4.1. BM-MSC Isolation and Expansion

Human bone samples from ND and D patients (*n* = 3 for each) (Table 1) undergoing knee joint replacement surgeries at Chapel Allerton Hospital, Leeds, were used for the isolation of human BM-MSCs. The samples were collected with ethical approval from the Yorkshire and Humberside National Research Ethics Committee (Reference number 14/YH/0087) and with the patients’ informed written consent.

Cancellous bony chips were obtained from the bone using sterile bone rongeurs, a dental chisel and a mallet and were incubated in a 1:1 mixture of 3 mg/mL collagenase type I (GIBCO™, Newcastle, Australia) and 4 mg/mL dispase (Roche, Basel, Switzerland) for 4 hrs at 37 °C with gentle agitation every 30 min, as previously described [73,74]. Digestion was arrested using 3 mLs of complete media: α Modified Eagle media (αMEM, Lonza BioWhittaker, Basel, Switzerland) supplemented with 20% foetal bovine serum, 1% penicillin/streptomycin and 1% L-glutamine (all from Sigma-Aldrich, St. Louis, MO, USA), and the cell suspension was aspirated away from the bony chips and centrifuged at 148× *g* for 5 min. The supernatant was removed, and the cell pellet was resuspended in complete media and passed through a 70 µm cell strainer (Falcon, Chandigarh, India). Cells were seeded in tissue culture flasks at a density of 4000 cells/cm^2^ for culture and expanded, passaged and cryopreserved as needed.

### 4.2. Colony-Forming Unit Fibroblast (CFU-F) Assay

Early P BM-MSCs (P 1–2) were counted and seeded at a density of 1000 cells per 100 mm tissue culture dish in duplicates. Media were changed every 5 days, and on day 14, media were removed. Cells were then gently washed twice with phosphate-buffered saline (PBS, Lonza BioWhittaker), fixed using 3.7% formaldehyde solution for 20 min and stained using 1% methylene blue in borate buffer solution for 45 min, as previously described [75]. The cells were gently washed with distilled water, and colonies of more than 50 cells were counted. The percentage of colony-forming MSCs for each assay was calculated using the equation, as previously described [76]:

% CFU-Fs = number of colonies/number of seeded cells × 100

### 4.3. Population Doubling Time Assay

P1 BM-MSCs were seeded in T25 flasks at a density of 2 × 10^5^ cells per flask. Cells were trypsinized using 0.25% (*w*/*v*) trypsin–EDTA solution (Sigma-Aldrich) when 80% confluent and counted. Counts of seeded and trypsinized cells, P numbers and passaging dates were recorded to generate a curve of accumulative population doublings vs. accumulative culture days, as previously described [77]. For each P, PD, APD and PDT were calculated using the following formulas:

PD = Log 2 (Count of trypsinized cells/count of seeded cells)

APD = Sum of PDs

PDT = Accumulative days of culture/APD

### 4.4. Flow Cytometry

Briefly, confluent cultures of BM-MSCs at P3-5 were trypsinized, counted, resuspended in PBS and incubated with fixable viability stain 780 (FVS 780, BD Biosciences, Plymouth, UK) at a concentration of 1:1000 (1 µL for each 1 mL of cells suspension) at room temperature for 10–15 min in the dark. Cells were then washed twice with PBS and resuspended in FACS buffer (PBS plus 0.5% *v*/*v* bovine serum albumin (BSA) and 0.05% *v*/*v* sodium azide). Cell suspension was aliquoted (each cell aliquot included approx. 1 million cells suspended in 50 µLs of FACS buffer), and Fc block reagent (0.5 mg/mL—BD Biosciences) was added to each tube (5 µL per tube) and incubated for 10 min at RT to reduce nonspecific binding. Brilliant stain buffer (BD Biosciences) was added to each of the stained tubes (50 µL per tube) to reduce the dye–dye interaction.

Cells were then incubated with anti-CD73-PE, anti-CD90-FITC, anti-CD105-BV421, anti-CD14-BV510, anti-CD19-APC, anti-CD34-BB700, anti-CD45-BV650 and anti-HLA-DR-BUV395 (all BD Biosciences used at concentrations recommended by manufacturer—see Table S3 in the Supplementary Data for further details) at 4 °C for 45 min in the dark, as previously described [32]. Unstained cells and fluorescence minus one (FMO)-stained cells were used as controls. Cells were washed twice, fixed using fixation buffer (BD Biosciences), resuspended in 500 µL of FACS buffer and kept at 4 °C until analysis within 1 week of the staining procedure. The analysis was performed using the CytoFLEX Lx flow cytometer (Beckman Coulter, High Wycombe, Buckinghamshire, UK), and the data were compensated and analysed using the CytExpert v 2.4 (Beckman Coulter) software. The compensation matrix was set up using BM-MSCs to compensate for the FVS 780 and single-stained compensation beads (BD Biosciences) for the remaining dyes in addition to unstained BM-MSCs and unstained beads serving as controls.

### 4.5. Osteogenic Differentiation of BM-MSCs

To assess the osteogenic potentials of BM-MSCs, cells at P 2–4 were cultured under basal conditions (complete media) and osteogenic conditions (complete media supplied with 10 nM dexamethasone, 50 µg/mL L-ascorbic acid and 5 mM β-glycerophosphate (all from Sigma-Aldrich)), as described previously [78], for 3 different durations: 1, 2 and 3 weeks.

For AR and ALP stains, cells were seeded in 6-well plates at a density of 3 × 10^4^ cells/ well. At each time point, cells were washed, fixed and stained as described in the next sections. For qPCR analysis, cells were seeded in T25 flasks at a density of 1 × 10^5^ cells. At each time point, cells were washed and lysed for RNA extraction and subsequent cDNA synthesis. At the experiment setup, 1 × 10^5^ of trypsinized cells were mixed with lysis buffer and frozen at −80 °C to serve as baseline (T0) controls.

#### 4.5.1. ALP Stain

At each time point, media were removed and BM-MSCs were washed gently twice with PBS, fixed using a 2:3 mixture of citrate working solution (Sigma-Aldrich) and acetone (VWR International, Radnor, PA, USA) for 30 s at RT. Fixative was washed and cells were stained with a Fast Blue dye mixture at RT for 30 min in the dark, as previously described [79,80].

#### 4.5.2. AR Staining and Quantification

AR staining was performed according to the manufacturer’s (ScienCell™ Research Laboratories, Carlsbad, CA, USA) instructions. At each time point, media were removed and BM-MSCs were washed gently twice with PBS and fixed using 4% formaldehyde solution for 15 min at RT. Fixative was removed and cells were washed with PBS and stained with AR stain for 20–30 min at RT with gentle shaking, as previously described [81].

AR stain quantification was performed according to the manufacturer’s (ScienCell™ Research Laboratories, Carlsbad, CA, USA) instructions. Briefly, stained cells were incubated with acetic acid for 30 min, detached and vortexed for 30 s. The solution was then heated at 85 °C for 10 min, centrifuged at 20,000× *g* for 15 min and 500 µL of the supernatant was transferred to a new tube. Next, 200 μL of 10% ammonium hydroxide was added to each tube to neutralize the acetic acid, and 150 μL of each sample was added in triplicate in an opaque-walled, transparent-bottomed 96-well plate (PerkinElmer, Waltham, MA, USA), as previously described [81]. The absorbance of each well was read at 405 nm using a Cytation 5 Imaging Plate Reader (BioTek, Winooski, VT, USA).

#### 4.5.3. Assessment of Gene Expression Using qPCR

RNeasy^®^ Mini Kit (Qiagen, Manchester, UK) was used for mRNA extraction including on-column gDNA digestion using an RNase-Free Dnase Set (Qiagen, UK) according to the manufacturer’s instructions. The quality of mRNA was assessed using A260/280 ratios to confirm the absence of contaminants and then mRNA was used for cDNA synthesis using a High-Capacity RNA-to-cDNA™ kit (Applied Biosystems, Warrington, UK) according to the manufacturer’s instructions, as previously described [82]. TaqMan^®^ Master Mix (Applied Biosystems, Warrington, UK), TaqMan^®^ probes (Applied Biosystems, UK—see Supplementary Data Table S2), cDNA and UltraPure™ DNase/RNase-Free distilled water (ThermoFisher Scientific, Oxford, UK) were mixed appropriately to conduct qPCR reactions in duplicate (a total of 20 µL per well) using a Roche 480 Light Cycler. Relative expression of periodontal (*COL1*, *POSTN* and *CEMP-1*), osteogenic (*ALPL*, *RUNX2* and *OCN*) and bone homeostasis (OPG and RANKL) marker genes were assessed. HPRT-1 was used as a housekeeping gene, as previously described [83], and the data were analysed using the ∆Ct method, where genes of interest were normalized to the housekeeping gene using the following equations:

∆Ct = (gene of interest − housekeeping gene)

The relative change in gene expression = 2^−∆Ct^

### 4.6. Statistical Analysis

The data were analysed using GraphPad Prism software v 9.2.0 (GraphPad Software). CFU-F assay, PDT assay, flow cytometry analysis and AR stain quantification data were presented as mean ± SD. qPCR data were presented as mean ± SEM. A paired *t* test was used to compare matched groups, and an unpaired *t* test was used to compare unmatched groups. RM-ANOVA was used to compare different time points. For all comparisons, *p* < 0.05 was considered significant.

## 5. Conclusions

While this in vitro study was limited by the relatively small sample size, the old age and the osteoarthritic comorbidity of all BM-MSC donors (which could have masked some of the osteogenic differentiation of both D and ND BM-MSCs), its main aim was to investigate the possible impact of T2DM on the osteogenic potentials of BM-MSCs, and it has shown that BM-MSCs from T2DM patients have comparable clonogenic and proliferative potentials, MSC immunophenotype and enumeration to nondiabetic controls. Although D and ND cells formed mineral deposits and expressed osteogenic markers similarly, D cells showed a lower response to osteogenic induction. D BM-MSCs showed lower expression levels of some key periodontal markers (POSTN and CEMP-1). The results of this study shed light on the possible effect of diabetes on the regeneration ability of BM-MSCs. However, further investigation into underlying mechanisms of POSTN and CEMP-1 lower expression levels in D BM-MSCs and how these molecules could be targeted to boost the regenerative potentials of these cells is warranted. Furthermore, future research using freshly isolated (native)BM-MSCs (to avoid epigenetic changes induced by cell culture [84]) and longer durations of osteogenic induction, as well as in vivo models to confirm bone and periodontal tissue regeneration following D BM-MSC transplantation, are required to further confirm the findings of the present study.

## Figures and Tables

**Table 1:** Demographic data of BM-MSC donors.

Donor	Age (yrs.)	Gender	Diabetic (HbA1C mmol/mol)	Other Comorbidities
D1	81	F	Yes [70]	OA, HT
D2	82	M	Yes (NA)	OA
D3	85	F	Yes [45]	OA
ND1	76	F	No	OA
ND2	64	M	No	OA
ND3	86	M	No	OA

D = diabetic, ND = nondiabetic, NA = not available, OA = osteoarthritis, HT = hypertension.

## Data Availability

Data will be made available upon request except for data that may compromise patients’ anonymity.

## Supplementary Materials

The following supporting information can be downloaded at: https://www.mdpi.com/article/10.3390/ijms25052851/s1.

## References

[1] Peng B.-Y., Dubey N.K., Mishra V.K., Tsai F.-C., Dubey R., Deng W.-P., Wei H.-J. (2018). Addressing stem cell therapeutic approaches in pathobiology of diabetes and its complications. J. Diabetes Res..

[2] Shanbhogue V.V., Hansen S., Frost M., Brixen K., Hermann A.P. (2017). Bone disease in diabetes: Another manifestation of microvascular disease?. Lancet Diabetes Endocrinol..

[3] Janssen J.A. (2021). Hyperinsulinemia and its pivotal role in aging, obesity, type 2 diabetes, cardiovascular disease and cancer. Int. J. Mol. Sci..

[4] Janssen J.A., Lamberts S.W. (2002). The role of IGF-I in the development of cardiovascular disease in type 2 diabetes mellitus: Is prevention possible?. Eur. J. Endocrinol..

[5] Louati K., Vidal C., Berenbaum F., Sellam J. (2015). Association between diabetes mellitus and osteoarthritis: Systematic literature review and meta-analysis. RMD Open.

[6] Eymard F., Parsons C., Edwards M., Petit-Dop F., Reginster J.-Y., Bruyère O., Richette P., Cooper C., Chevalier X. (2015). Diabetes is a risk factor for knee osteoarthritis progression. Osteoarthr. Cartil..

[7] Kurra S., Siris E. (2014). Diabetes and bone health: The relationship between diabetes and osteoporosis-associated fractures. Diabetes Metab. Res. Rev..

[8] Kinane D.F., Stathopoulou P.G., Papapanou P.N. (2017). Periodontal diseases. Nat. Rev. Dis. Primers.

[9] Dimitriou R., Jones E., McGonagle D., Giannoudis P.V. (2011). Bone regeneration: Current concepts and future directions. BMC Med..

[10] Bosshardt D.D., Sculean A. (2009). Does periodontal tissue regeneration really work?. Periodontology 2000.

[11] Bassir S.H., Wisitrasameewong W., Raanan J., Ghaffarigarakani S., Chung J., Freire M., Andrada L.C., Intini G. (2016). Potential for Stem Cell-Based Periodontal Therapy. J. Cell. Physiol..

[12] Yousefi A.M., James P.F., Akbarzadeh R., Subramanian A., Flavin C., Oudadesse H. (2016). Prospect of Stem Cells in Bone Tissue Engineering: A Review. Stem Cells Int..

[13] Mohamed-Ahmed S., Fristad I., Lie S.A., Suliman S., Mustafa K., Vindenes H., Idris S.B. (2018). Adipose-derived and bone marrow mesenchymal stem cells: A donor-matched comparison. Stem Cell Res. Ther..

[14] Bartold M., Gronthos S., Haynes D., Ivanovski S. (2019). Mesenchymal stem cells and biologic factors leading to bone formation. J. Clin. Periodontol..

[15] Dadras M., May C., Wagner J.M., Wallner C., Becerikli M., Dittfeld S., Serschnitzki B., Schilde L., Guntermann A., Sengstock C. (2020). Comparative proteomic analysis of osteogenic differentiated human adipose tissue and bone marrow-derived stromal cells. J. Cell. Mol. Med..

[16] Lyu J., Hashimoto Y., Honda Y., Matsumoto N. (2021). Comparison of osteogenic potentials of dental pulp and bone marrow mesenchymal stem cells using the new cell transplantation platform, cellsaic, in a rat congenital cleft-jaw model. Int. J. Mol. Sci..

[17] Chahal J., Gómez-Aristizábal A., Shestopaloff K., Bhatt S., Chaboureau A., Fazio A., Chisholm J., Weston A., Chiovitti J., Keating A. (2019). Bone Marrow Mesenchymal Stromal Cell Treatment in Patients with Osteoarthritis Results in Overall Improvement in Pain and Symptoms and Reduces Synovial Inflammation. Stem Cells Transl. Med..

[18] Lamo-Espinosa J.M., Mora G., Blanco J.F., Granero-Moltó F., Núñez-Córdoba J.M., López-Elío S., Andreu E., Sánchez-Guijo F., Aquerreta J.D., Bondía J.M. (2018). Intra-articular injection of two different doses of autologous bone marrow mesenchymal stem cells versus hyaluronic acid in the treatment of knee osteoarthritis: Long-term follow up of a multicenter randomized controlled clinical trial (phase I/II). J. Transl. Med..

[19] Al-Najar M., Khalil H., Al-Ajlouni J., Al-Antary E., Hamdan M., Rahmeh R., Alhattab D., Samara O., Yasin M., Al Abdullah A. (2017). Intra-articular injection of expanded autologous bone marrow mesenchymal cells in moderate and severe knee osteoarthritis is safe: A phase I/II study. J. Orthop. Surg. Res..

[20] Shin Y.S., Yoon J.R., Kim H.S., Lee S.H. (2018). Intra-articular Injection of bone marrow-derived mesenchymal stem cells leading to better clinical outcomes without difference in MRI outcomes from baseline in patients with knee osteoarthritis. Knee Surg. Relat. Res..

[21] Dawson J.I., Kanczler J., Tare R., Kassem M., Oreffo R.O.C. (2014). Concise review: Bridging the gap: Bone regeneration using skeletal stem cell-based strategies-where are we now?. Stem Cells.

[22] Baba S., Yamada Y., Komuro A., Yotsui Y., Umeda M., Shimuzutani K., Nakamura S. (2016). Phase I/II Trial of Autologous Bone Marrow Stem Cell Transplantation with a Three-Dimensional Woven-Fabric Scaffold for Periodontitis. Stem Cells Int..

[23] Mahmoud M., Abu-Shahba N., Azmy O., El-Badri N. (2019). Impact of Diabetes Mellitus on Human Mesenchymal Stromal Cell Biology and Functionality: Implications for Autologous Transplantation. Stem Cell Rev. Rep..

[24] Andrzejewska A., Catar R., Schoon J., Qazi T.H., Sass F.A., Jacobi D., Blankenstein A., Reinke S., Krüger D., Streitz M. (2019). Multi-Parameter Analysis of Biobanked Human Bone Marrow Stromal Cells Shows Little Influence for Donor Age and Mild Comorbidities on Phenotypic and Functional Properties. Front. Immunol..

[25] Cassidy F.C., Shortiss C., Murphy C.G., Kearns S.R., Curtin W., De Buitléir C., O’brien T., Coleman C.M. (2020). Impact of Type 2 Diabetes Mellitus on Human Bone Marrow Stromal Cell Number and Phenotypic Characteristics. Int. J. Mol. Sci..

[26] Sun R., Liang C., Sun Y., Xu Y., Geng W., Li J. (2021). Effects of metformin on the osteogenesis of alveolar BMSCs from diabetic patients and implant osseointegration in rats. Oral Dis..

[27] Pi S.H., Lee S.K., Hwang Y.S., Choi M.G., Lee S.K., Kim E.C. (2007). Differential expression of periodontal ligament-specific markers and osteogenic differentiation in human papilloma virus 16-immortalized human gingival fibroblasts and periodontal ligament cells. J. Periodontal. Res..

[28] Du J., Li M. (2017). Functions of Periostin in dental tissues and its role in periodontal tissues’ regeneration. Cell. Mol. Life Sci..

[29] Hoz L., Romo E., Zeichner-David M., Sanz M., Nuñez J., Gaitán L., Mercado G., Arzate H. (2012). Cementum protein 1 (CEMP1) induces differentiation by human periodontal ligament cells under three-dimensional culture conditions. Cell Biol. Int..

[30] Hoz L., López S., Zeichner-David M., Arzate H. (2021). Regeneration of rat periodontium by cementum protein 1-derived peptide. J. Periodontal Res..

[31] Dominici M., Le Blanc K., Mueller I., Slaper-Cortenbach I., Marini F.C., Krause D.S., Deans R.J., Keating A., Prockop D.J., Horwitz E.M. (2006). Minimal criteria for defining multipotent mesenchymal stromal cells. The International Society for Cellular Therapy position statement. Cytotherapy.

[32] Alkharobi H., Beattie J., Meade J., Devine D., El-Gendy R. (2017). Dental Pulp Cells Isolated from Teeth with Superficial Caries Retain an Inflammatory Phenotype and Display an Enhanced Matrix Mineralization Potential. Front. Physiol..

[33] Flouzat-Lachaniette C.H., Heyberger C., Bouthors C., Roubineau F., Chevallier N., Rouard H., Hernigou P. (2016). Osteogenic progenitors in bone marrow aspirates have clinical potential for tibial non-unions healing in diabetic patients. Int. Orthop..

[34] Brewster L., Robinson S., Wang R., Griffiths S., Li H., Peister A., Copland I., McDevitt T. (2017). Expansion and angiogenic potential of mesenchymal stem cells from patients with critical limb ischemia. J. Vasc. Surg..

[35] Siegel G., Kluba T., Hermanutz-Klein U., Bieback K., Northoff H., Schäfer R. (2013). Phenotype, donor age and gender affect function of human bone marrow-derived mesenchymal stromal cells. BMC Med..

[36] Stolzing A., Jones E., McGonagle D., Scutt A. (2008). Age-related changes in human bone marrow-derived mesenchymal stem cells: Consequences for cell therapies. Mech. Ageing Dev..

[37] Gabr M.M., Zakaria M.M., Refaie A.F., Ismail A.M., Abou-El-Mahasen M.A., Ashamallah S.A., Khater S.M., El-Halawani S.M., Ibrahim R.Y., Uin G.S. (2013). Insulin-producing cells from adult human bone marrow mesenchymal stem cells control streptozotocin-induced diabetes in nude mice. Cell Transplant..

[38] Chang T.C., Hsu M.F., Wu K.K. (2015). High glucose induces bone marrow-derived mesenchymal stem cell senescence by upregulating autophagy. PLoS ONE.

[39] Bian Y., Ma X., Wang R., Yuan H., Chen N., Du Y. (2019). Human amnion-derived mesenchymal stem cells promote osteogenesis of human bone marrow mesenchymal stem cells against glucolipotoxicity. FEBS Open Bio.

[40] Lu Y.Q., Lu Y., Li H.J., Cheng X.B. (2012). Effect of advanced glycosylation end products (AGEs) on proliferation of human bone marrow mesenchymal stem cells (MSCs) in vitro. In Vitro Cell. Dev. Biol. Anim..

[41] Dohl J., Foldi J., Heller J., Gasier H.G., Deuster P.A., Yu T. (2018). Acclimation of C2C12 myoblasts to physiological glucose concentrations for in vitro diabetes research. Life Sci..

[42] Ribot J., Denoeud C., Frescaline G., Landon R., Petite H., Pavon-Djavid G., Bensidhoum M., Anagnostou F. (2021). Experimental type 2 diabetes differently impacts on the select functions of bone marrow-derived multipotent stromal cells. Cells.

[43] Liang C., Liu X., Liu C., Xu Y., Geng W., Li J. (2022). Integrin α10 regulates adhesion, migration, and osteogenic differentiation of alveolar bone marrow mesenchymal stem cells in type 2 diabetic patients who underwent dental implant surgery. Bioengineered.

[44] Zhang P., Zhang H., Lin J., Xiao T., Xu R., Fu Y., Zhang Y., Du Y., Cheng J., Jiang H. (2020). Insulin impedes osteogenesis of BMSCs by inhibiting autophagy and promoting premature senescence via the TGF-β1 pathway. Aging.

[45] Franke S., Rüster C., Pester J., Hofmann G., Oelzner P., Wolf G. (2011). Advanced glycation end products affect growth and function of osteoblasts. Clin. Exp. Rheumatol..

[46] Açil Y., Ghoniem A.A., Gülses A., Kisch T., Stang F., Wiltfang J., Gierloff M. (2017). Suppression of osteoblast-related genes during osteogenic differentiation of adipose tissue derived stromal cells. J. Cranio-Maxillofac. Surg..

[47] Abuarqoub D., Zaza R., Aslam N., Jafar H., Zalloum S., Atoom R., Awidi A. (2021). The Role of Biodentine^TM^ on the Odontogenic/Osteogenic Differentiation of Human Dental Pulp Stem Cells. Appl. Sci..

[48] Wang Q.-N., Yan Y.-Z., Zhang X.-Z., Lv J.-X., Nie H.-P., Wu J., Wu D., Yuan S.-S., Tang C.-B. (2022). Rescuing effects of periostin in advanced glycation end-products (AGEs) caused osteogenic and oxidative damage through AGE receptor mediation and DNA methylation of the CALCA promoter. Chem. Biol. Interact..

[49] Seubbuk S., Sritanaudomchai H., Kasetsuwan J., Surarit R. (2017). High glucose promotes the osteogenic differentiation capability of human periodontal ligament fbroblasts. Mol. Med. Rep..

[50] Merle B., Garnero P. (2012). The multiple facets of periostin in bone metabolism. Osteoporos. Int..

[51] Wu Z., Dai W., Wang P., Zhang X., Tang Y., Liu L., Wang Q., Li M., Tang C. (2018). Periostin promotes migration, proliferation, and differentiation of human periodontal ligament mesenchymal stem cells. Connect. Tissue Res..

[52] Mohanarangam S., Victor D.J., Subramanian S., Prakash PS G. (2021). The influence of periostin on osteoblastic adhesion and proliferation on collagen matrices—An in vitro study. J. Indian Soc. Periodontol..

[53] Yan Y., Zhang H., Liu L., Chu Z., Ge Y., Wu J., Liu Y., Tang C. (2020). Periostin reverses high glucose-inhibited osteogenesis of periodontal ligament stem cells via AKT pathway. Life Sci..

[54] Aida Y., Kurihara H., Kato K. (2018). Wnt3a promotes differentiation of human bone marrow-derived mesenchymal stem cells into cementoblast-like cells. In Vitro Cell. Dev. Biol. Anim..

[55] Kim J.E., Kim T.G., Lee Y.H., Yi H.K. (2020). Phelligridin D maintains the function of periodontal ligament cells through autophagy in glucose-induced oxidative stress. J. Periodontal Implant. Sci..

[56] Gokhan K., Keklikoglu N., Buyukertan M. (2004). The comparison of the thickness of the cementum layer in type 2 diabetic and non-diabetic patients. J. Contemp. Dent. Pract..

[57] Carmona-Rodríguez B., Álvarez-Pérez M.A., Narayanan A.S., Zeichner-David M., Reyes-Gasga J., Molina-Guarneros J., García-Hernández A.L., Suárez-Franco J.L., Gil Chavarría I., Villarreal-Ramírez E. (2007). Human Cementum Protein 1 induces expression of bone and cementum proteins by human gingival fibroblasts. Biochem. Biophys. Res. Commun..

[58] Arroyo R., López S., Romo E., Montoya G., Hoz L., Pedraza C., Garfias Y., Arzate H. (2020). Carboxy-terminal cementum protein 1-derived peptide 4 (Cemp1-p4) promotes mineralization through wnt/β-catenin signaling in human oral mucosa stem cells. Int. J. Mol. Sci..

[59] Ying X., Chen X., Liu H., Nie P., Shui X., Shen Y., Yu K., Cheng S. (2015). Silibinin alleviates high glucose-suppressed osteogenic differentiation of human bone marrow stromal cells via antioxidant effect and PI3K/Akt signaling. Eur. J. Pharmacol..

[60] Deng X., Xu M., Shen M., Cheng J. (2018). Effects of Type 2 Diabetic Serum on Proliferation and Osteogenic Differentiation of Mesenchymal Stem Cells. J. Diabetes Res..

[61] Liu W., Konermann A., Guo T., Jäger A., Zhang L., Jin Y. (2014). Canonical Wnt signaling differently modulates osteogenic differentiation of mesenchymal stem cells derived from bone marrow and from periodontal ligament under inflammatory conditions. Biochim. Biophys. Acta.

[62] Vimalraj S. (2020). Alkaline phosphatase: Structure, expression and its function in bone mineralization. Gene.

[63] Mostafa N.Z., Fitzsimmons R., Major P.W., Adesida A., Jomha N., Jiang H., Uludağ H. (2012). Osteogenic differentiation of human mesenchymal stem cells cultured with dexamethasone, vitamin D3, basic fibroblast growth factor, and bone morphogenetic protein-2. Connect. Tissue Res..

[64] Komori T. (2018). Runx2, an inducer of osteoblast and chondrocyte differentiation. Histochem. Cell Biol..

[65] Boyce B.F., Xing L. (2008). Functions of RANKL/RANK/OPG in bone modeling and remodeling. Arch. Biochem. Biophys..

[66] Zhang L., Ding Y., Rao G.Z., Miao D. (2016). Effects of IL-10 and glucose on expression of OPG and RANKL in human periodontal ligament fibroblasts. Braz. J. Med. Biol. Res..

[67] Wu Y., Liu F., Zhang X., Shu L. (2014). Insulin modulates cytokines expression in human periodontal ligament cells. Arch. Oral Biol..

[68] Feng Y., Liu J., Liu H. (2012). AMP-activated protein kinase acts as a negative regulator of high glucose-induced RANKL expression in human periodontal ligament cells. Chin. Med. J..

[69] Zheng J., Chen S., Albiero M.L., Vieira G.H.A., Wang J., Feng J.Q., Graves D.T. (2018). Diabetes Activates Periodontal Ligament Fibroblasts via NF-κB In Vivo. J. Dent. Res..

[70] Churchman S.M., Ponchel F., Boxall S.A., Cuthbert R., Kouroupis D., Roshdy T., Giannoudis P.V., Emery P., McGonagle D., Jones E.A. (2012). Transcriptional profile of native CD271+ multipotential stromal cells: Evidence for multiple fates, with prominent osteogenic and Wnt pathway signaling activity. Arthritis Rheum..

[71] Suliman S., Ali H.R., Karlsen T.A., Amiaud J., Mohamed-Ahmed S., Layrolle P., Costea D.E., Brinchmann J.E., Mustafa K. (2019). Impact of humanised isolation and culture conditions on stemness and osteogenic potential of bone marrow derived mesenchymal stromal cells. Sci. Rep..

[72] Bhattarai G., Min C.K., Jeon Y.M., Bashyal R., Poudel S.B., Kook S.H., Lee J.C. (2019). Oral supplementation with p-coumaric acid protects mice against diabetes-associated spontaneous destruction of periodontal tissue. J. Periodontal Res..

[73] Campbell T.M., Churchman S.M., Gomez A., McGonagle D., Conaghan P.G., Ponchel F., Jones E. (2016). Mesenchymal Stem Cell Alterations in Bone Marrow Lesions in Patients with Hip Osteoarthritis. Arthritis Rheumatol..

[74] Ilas D.C., Baboolal T.G., Churchman S.M., Jones W.G., Giannoudis P.V., Bühring H.-J., McGonagle D., Jones E. (2020). The osteogenic commitment of CD271+CD56+ bone marrow stromal cells (BMSCs) in osteoarthritic femoral head bone. Sci. Rep..

[75] Sanjurjo-Rodriguez C., Baboolal T.G., Burska A.N., Ponchel F., El-Jawhari J.J., Pandit H., McGonagle D., Jones E. (2019). Gene expression and functional comparison between multipotential stromal cells from lateral and medial condyles of knee osteoarthritis patients. Sci. Rep..

[76] Russell T., Watad A., Bridgewood C., Rowe H., Khan A., Rao A., Loughenbury P., Millner P., Dunsmuir R., Cuthbert R. (2021). Il-17a and TNF modulate normal human spinal entheseal bone and soft tissue mesenchymal stem cell osteogenesis, adipogenesis, and stromal function. Cells.

[77] Owston H.E., Ganguly P., Tronci G., Russell S.J., Giannoudis P.V., Jones E.A. (2019). Colony Formation, Migratory, and Differentiation Characteristics of Multipotential Stromal Cells (MSCs) from “Clinically Accessible” Human Periosteum Compared to Donor-Matched Bone Marrow MSCs. Stem Cells Int..

[78] Etheridge S.L., Spencer G.J., Heath D.J., Genever P.G. (2004). Expression Profiling and Functional Analysis of Wnt Signaling Mechanisms in Mesenchymal Stem Cells. Stem Cells.

[79] Huang D.-M., Chung T.-H., Hung Y., Lu F., Wu S.-H., Mou C.-Y., Yao M., Chen Y.-C. (2008). Internalization of mesoporous silica nanoparticles induces transient but not sufficient osteogenic signals in human mesenchymal stem cells. Toxicol. Appl. Pharmacol..

[80] Hsiao J., Tsai C., Chung T., Hung Y., Yao M., Liu H., Mou C., Yang C., Chen Y., Huang D. (2008). Mesoporous silica nanoparticles as a delivery system of gadolinium for effective human stem cell tracking. Small.

[81] Gregory C.A., Gunn W.G., Peister A., Prockop D.J. (2004). An Alizarin red-based assay of mineralization by adherent cells in culture: Comparison with cetylpyridinium chloride extraction. Anal. Biochem..

[82] Alkharobi H., Alhodhodi A., Hawsawi Y., Alkafaji H., Devine D., El-Gendy R., Beattie J. (2016). IGFBP-2 and -3 co-ordinately regulate IGF1 induced matrix mineralisation of differentiating human dental pulp cells. Stem Cell Res..

[83] Amable P.R., Teixeira M.V.T., Carias R.B.V., Granjeiro J.M., Borojevic R. (2013). Identification of appropriate reference genes for human mesenchymal cells during expansion and differentiation. PLoS ONE.

[84] Ghazanfari R., Zacharaki Di Li H., Ching Lim H., Soneji S., Scheding S. (2017). Human Primary Bone Marrow Mesenchymal Stromal Cells and Their in vitro Progenies Display Distinct Transcriptional Profile Signatures. Sci. Rep..

