# Supplementary material for: Characterisation and Expression of Osteogenic and Periodontal Markers of Bone Marrow Mesenchymal Stem Cells (BM-MSCs) from Diabetic Knee Joints
Source: Int J Mol Sci. 2024 Mar 1;25(5):2851. doi: 10.3390/ijms25052851 (PMC10931945; doi:10.3390/ijms25052851)
Supplement: Supplementary file 1 [file ijms-25-02851-s001.zip › ijms-2833057-supplementary.pdf]

Table S1: PD, APD and PDT of non-diabetic (ND) and diabetic (D) BM-MSCs

Passage	*PD*		*p value*	*APD*		*p value*	*PDT*		*p value*
	ND (mean ± SD)	*D (mean ± SD)*		*ND (mean ± SD)*	*D (mean ± SD)*		*ND (mean ± SD)*	*D (mean ± SD)*	
P2	*1.72 ± 0.38*	*1.33 ± 0.79*	*0.5*	*2.72 ± 0.99*	*2.74 ± 0.54*	*0.97*	*8.1 ± 0.34*	*5.82 ± 1.4*	*0.1*
P3	*1.49 ± 0.89*	*1.49 ± 0.66*	*0.99*	*4.21 ± 1.73*	*4.23 ± 1.18*	*0.99*	*8.45 ± 1.56*	*6.24 ± 0.58*	*0.12*
P4	*0.87 ± 0.51*	*1.31 ± 0.54*	*0.37*	*5.08 ± 2.22*	*5.54 ± 1.69*	*0.79*	*11.28 ± 3.56*	*6.58 ± 1.31*	*0.14*
P5	*0.67 ± 0.72*	*0.92 ± 0.34*	*0.63*	*5.76 ± 2.93*	*6.46 ± 1.93*	*0.75*	*12.48 ± 4.79*	*7.91 ± 2.4*	*0.24*

APD: accumulative population doubling, PD: population doubling, PDT: population doubling time.

TableS2: List of TaqMan® gene expression assays used in qPCR.

#	Gene	Description (reference)	TaqMan® Gene Expression Assay number
1	*Hypoxanthine-guanine phosphoribosyl transferase 1 (HPRT1)*	*Housekeeping gene*	*Hs99999909_m1*
2	*Alkaline phosphatase (ALPL)*	*Osteogenic marker*	*Hs01029144-m1*
3	*Runt-related transcription factor-2 (RUNX2)*	*Transcription factor - Early marker of osteogenic differentiation*	*Hs00231692-m1*
4	*Osteocalcin (OCN)*	*Calcium binding ECM protein - Late marker of osteogenic differentiation*	*Hs00609452-g1*
5	*Collagen1 A1 (COL1A1)*	*Marker of fibroblasts and osteoblasts differentiation*	*Hs00164004_m1*
6	*Periostin (POSTN)*	*Marker of PDL fibroblasts differentiation*	*Hs01566750_m1*
7	*Cementum protein-1 (CEMP-1)*	*Marker of cementoblasts differentiation*	*Hs04185363_s1*
8	*Osteoprotegerin (OPG-TNFRSF11B)*	*Receptor for TNF and decoy receptor for RANKL*	*Hs00900360_m1*
9	*Receptor activator NF-κβ ligand (RANKL)*	*Marker of osteoclasts differentiation and bone resorption*	*Hs01092186_m1*

TableS3: Antibodies used in flow cytometry analysis

Antibody against	Fluorophore	Clone	Product number	Stock concentration	Working concentration	Manufacturer
CD73	Phycoerythrin (PE)	AD2	561014	6.3 µg/mL	0.44 µg/mL	BD Biosciences
CD90	Fluorescein Isothiocyanate (FITC)	5E10	561969	0.5 mg/mL	1.7 µg/mL	
CD105	Brilliant Violet 421 (BV421)	266	566265	100 µg/mL	3.46 µg/mL	
CD14	Brilliant Violet 510 (BV510)	MØP9	563079	100 µg/mL	0.69 µg/mL	
CD19	Allophycocyanine (APC)	HIB19	561742	6.3 µg/mL	0.87 µg/mL	
CD34	Brilliant Blue 700 (BB700)	581	745835	0.2 mg/mL	0.69 µg/mL	
CD45	Brilliant Violet 650 (BV650)	HI30	563717	100 µg/mL	1.73 µg/mL	
HLA-DR	Brilliant Ultraviolet 395 (BUV395)	G46-6	565972	200 µg/mL	6.92 µg/mL	
